# Yeast Peptides Improve the Intestinal Barrier Function and Alleviate Weaning Stress by Changing the Intestinal Microflora Structure of Weaned Lambs

**DOI:** 10.3390/microorganisms11102472

**Published:** 2023-10-01

**Authors:** Yanjun Li, Lulu Han, Jie Liu, Lingyun Kang, Ling Zhao, Kai Cui

**Affiliations:** 1 National Key Laboratory of Agricultural Microbiology, Huazhong Agricultural University, Wuhan 430070, China; lyjyhr@126.com; 2 Key Laboratory of Feed Biotechnology of the Ministry of Agriculture and Rural Affairs, Institute of Feed Research of Chinese Academy of Agricultural Sciences, Beijing 100081, China; joyce_han1998@163.com (L.H.); liujie830821@126.com (J.L.); 18821622269@163.com (L.K.)

**Keywords:** weaning stress, lamb, yeast peptide, microbiota

## Abstract

Early weaning stress in lambs leads to decreased feed intake, damage to intestinal morphology, changes in the microbial flora structure, and subsequent complications. Yeast peptides are antimicrobial peptides with anti-inflammatory, antioxidant, and bacteriostasis effects. To study the effects of yeast peptides on relieving weaning stress in lambs, 54 lambs were randomly divided into three groups: ewe-reared (ER), yeast-peptide-treated (AP), and early-weaned (EW) lambs. The body weight and dry matter intake did not significantly differ among all groups. After weaning, the daily gain and feed conversion rate decreased significantly (*p* < 0.01), but AP showed an upward trend. In the EW group, immunoglobulin (Ig) levels changed significantly post-weaning (IgG decreased; IgA and IgM increased); the villi shortened, the crypt depth increased, and the villi height/crypt depth decreased (*p* < 0.001). The abundance and diversity of microflora among all groups were not significantly different. A column coordinate analysis showed significant differences in the intestinal microbial structure between the AP and EW groups. *Lactobacillus*, *Aeriscardovia*, *Ruminosaceae_UCG-014*, and *Catenisphaera* may play key roles in alleviating weaning stress in lambs. Our study provides new clues for alleviating weaning stress in lambs by describing the influence of yeast peptides on the intestinal microflora during weaning.

## 1. Introduction

In recent years, because of resources and environmental restrictions, house feeding and intensive breeding have become major trends in the sheep industry [1]. Early weaning methods have become common practice in lamb breeding in many countries to improve the utilization rate of ewes [1]. However, early weaning requires transitioning the lambs from milk to solid feed as a source of nutrition. During this process, the intestinal tract of the lamb develops rapidly, but is not yet optimal, and its ability to digest and absorb feed is inefficient. Therefore, weaning, a crucial event in the early development of animals, induces a stress response that leads to decreased feed intake, abnormal mucosal immunity, and increased mortality in young animals [2,3,4].

The small intestine, an important multifunctional organ, plays essential roles in the digestion and absorption of nutrients, regulation of immune responses, and secretion of various enzymes and immunoglobulins [5,6]. However, after weaning, young ruminants experience a series of microbial colonizations, exogenous pathogen invasions, and maternal antibody losses, which induce significant changes to their intestinal structure and function [5,7,8]. This is crucial for lambs with immature digestive and immune systems [9]. The gastrointestinal microbiota in ruminants is one of the main indicators of healthy animal development and productivity. The intestinal microbiota plays a key role in host health by providing metabolites, maintaining metabolic functions, developing the immune system, and resisting pathogens [10,11]. Studies have shown that the intestinal microbiota of young ruminants is easily affected by the external environment, and weaning stress can significantly affect the diversity of intestinal microbiota, thus inducing an inflammatory response and disrupting intestinal homeostasis [12]. Therefore, the alleviation of weaning stress in lambs by regulating the structure of the intestinal microbiota is a hot topic.

Bioactive peptides are protein fragments consisting of 3–20 amino acids, and their biological activity is closely related to the composition, size, sequences, and physicochemical properties of these amino acids [13]. Yeast peptides represent a type of bioactive peptide which can be generated by various yeast strains. The primary source is *Saccharomyces cerevisiae* fermentation [13], although occasionally, they can also be produced by *Kluyvermyces marxianus* [14] and *Candida utilities* [15]. Yeast peptides have the advantages of fast reproduction, a short culture cycle, low cost, strong antibacterial ability, no drug resistance, no residue, high-temperature resistance, acid and alkali resistance, and enzyme resistance [16,17]. Yeast peptides have been widely used and studied in ruminant breeding. The diversity of yeast polypeptide chains can satisfy all complex physiological regulatory functions of organisms, such as antioxidation [18,19], anti-inflammation [19], anti-hypertension [20], anti-diabetes [15], anti-ulcer [21], and bacteriostasis functionsm, among others [13].

In this study, we aimed to explore the effects of early weaning and yeast peptide addition on the jejunal microbiota of 18 lambs using 16S rDNA technology. Our study provides a theoretical basis for exploring the intestinal nutrient regulators and improving the intestinal microbiota of weaned lambs.

## 2. Materials and Methods

### 2.1. Study Regions, Animals, and Experimental Design

This study was conducted at the Linqing Runlin Animal Husbandry Corporation, Ltd. (115°83′ E, 36°82′ N) in Liaocheng City, Shandong Province, China. In October 2020, a total of 54 neonatal healthy Hu lambs born from double pregnancy with almost the same birth weight (3.58 ± 0.59 kg) were selected for the experiment and randomly divided into three groups. One group was ewe reared (ER) during the whole trial (35 days), whereas the other two groups were weaned and separated from their dams at 28 days. In these two early weaning groups, one group was fed 1 g/day yeast peptide from day 21 until the end of the experiment (AP), and the other group did not receive any treatment besides weaning (EW).

The experimental region was a semi-open sheep house with good lighting and ventilation. From birth to 28 days, all lambs were reared with ewes in the same pen and began to consume the same starter feed on day 7. The ingredients and chemical composition of the pelleted starter feed (pellet size: 3.2 mm diameter and 8 mm length) are listed in Table 1. The yeast peptide provided by Beijing Enhalor International Tech Co., Ltd., Beijing, China is a 19-amino-acid-long antimicrobial peptide (GGVGKIIEYFIGGGVGRYG) produced via a yeast fermentation process. The composition of the yeast peptide is crude protein (≥3%) and mannosan (0.5%) and its concentration is 5000 mg/kg. There were three ewes and their lambs (*n* = 6) in each pen, and each group had three pens. All ewes were fed twice daily according to the farm’s feeding management schedule. In addition, no lambs in the groups could get to the ewes’ feed. The ER lambs were kept in the same pen as their ewes during the entire experimental period. However, the ewes in the EW and AP groups were removed on day 28, and the lambs were left in the original pen and weaned by one-time abstinence. From day 21 to day 35, the lambs in the AP group were fed yeast peptide at 18:00 every day. All lambs had free access to starter feed and water throughout the whole experiment.

### 2.2. Feed Intake and Growth Performance Measurement

The body weight (BW) of all lambs was measured on days 21, 28, and 35 and thereafter before the morning feeding during the experimental period. The average daily gain (ADG) of each lamb was calculated according to the BW at each stage. From the starter supplied to each pen, the dry matter intake (DMI) of the starter feed for the lambs was calculated, and the remaining and added amounts of the starter feed were accurately weighed and recorded at 08:00 every day. The average daily feed intake of lambs in each pen was calculated and recorded as the DMI of lambs in each pen. The feed efficiency of each group was calculated as ADG/DMI.

### 2.3. Blood Sample Collection and Measurement

On days 21, 28, 31, and 35 of the experiment, six lambs in each group with similar body weights were selected to collect blood samples of approximately 5 mL by jugular vein puncture before the morning feeding. Isolated serum was obtained from the blood sample after centrifuging at 1500× *g* for 30 min at 4 °C, and was stored in 1.5 mL cryotubes after that at −20 °C until further analysis.

Serum immunoglobulin A (IgA), immunoglobulin G (IgG), and immunoglobulin M (IgM) concentrations were analyzed using an automatic biochemical analyzer (Kehua-zy KHB-1280, Shanghai, China) with the corresponding commercial test kits. The specifications and models of the experimental kits are as follows: IgG (No. JH-00013), IgA (No. JH-00014), and IgM (No. JH-00015) purchased from Beijing Jinhai Keyu Biotechnology Development Co., Ltd., Beijing, China (coefficient of variation < 5%).

### 2.4. Jejunum Sampling, Processing, and Histomorphology Analysis

After blood sampling, six lambs from each group were slaughtered on day 35. Immediately after slaughter, the contents of the jejunum were quickly sampled in 2 mL sterile lyophilized tubes and were snap-frozen in liquid nitrogen and subsequently stored at −80 °C for further microbial analysis. The middle part of the jejunum was sampled and washed with saline to remove the chyme of the samples then fixed in 4% paraformaldehyde (Sigma-Aldrich, St. Louis, MO, USA) for analysis of intestinal morphology. Thereafter, sections were trimmed from each sample by a transverse cut through the villi, dehydrated with an ethanol and toluene series (Beijing Chemical Works, Beijing, China), and embedded in paraffin (Leica Microsystems GmbH, Wetzlar, Germany). After embedding, a series of sections (5 μm thickness) were cut using a paraffin slicing machine (RM2016, Laika Ltd., Shanghai, China) and mounted on gelatin-coated glass slides and stained with hematoxylin and eosin (H&E). Finally, the villi height, crypt depth, and villi height/crypt depth (V/C) were determined for each section using Digital Pathology Viewer software (v 2.0.4.0104, Shenzhen Shengqiang Technology Co., Ltd., Shenzhen, China).

### 2.5. DNA Extraction, PCR Amplification, and 16S rRNA Sequencing

The process of obtaining microbial DNA from jejunal content samples (*n* = 18) was meticulously carried out using a PowerSoil DNA Isolation Kit (Mo Bio Laboratories, Carlsbad, CA, USA) following precise extraction protocols. To ensure the quality of DNA, its concentration and purity were meticulously scrutinized using a Thermo NanoDrop 2000 Spectrophotometer (Thermo Fisher Scientific, Waltham, MA, USA). Additionally, we employed 1% agarose gels for a comprehensive quality assessment. The next step in our methodology involved the targeted amplification of the V3-V4 region of the bacterial 16S rRNA gene. This was achieved by utilizing the extracted bacterial DNA as a template and employing a specific primer pair: 338F (5′-ACTCCTACGGGAGGCAGCAG-3′) and 806R (5′-GGACTACHVGGGTWTCTAAT-3′) [22]. The PCR amplification procedure was initiated with an initial denaturation step at 94 °C for 5 min, followed by 28 cycles of amplification, which consisted of denaturation at 94 °C for 45 s, annealing at 55 °C for 30 s, and extension at 72 °C for 45 s. The process was concluded with a final extension step at 72 °C for 10 min. All PCR reactions were carried out with a reaction volume of 25 µL, in accordance with the method established by HAN et al. [23]. Following amplification, the recovered PCR products underwent rigorous examination and confirmation by 2% agarose gel electrophoresis. Notably, throughout these critical steps, both negative and positive controls were rigorously implemented for quality control. The results unequivocally demonstrated that any potential contamination was negligible and had no discernible impact on the biological variation observed among the distinct experimental groups. Following confirmation of the amplified products, the subsequent purification step employed an Agencourt AMPure XP kit (Beckman Coulter, La Brea, CA, USA), in strict adherence to the manufacturer’s instructions. Quantification of the purified amplicons was meticulously performed using QuantiFluor-ST (Promega, Madison, WI, USA). Finally, to unlock the genetic information encapsulated in the purified amplicons, we subjected them to sequencing on an Illumina MiSeq-PE300 platform (Illumina Inc., San Diego, CA, USA), generating high-quality 2 × 300 bp paired-end reads.

### 2.6. Sequence Analysis

Subsequently, the paired-end reads underwent a merging process facilitated by Flash (version 1.20) [24]. Each sample was then demultiplexed based on its unique barcode. The original sequencing data were meticulously secured after the removal of barcodes, primers, and splice variants. To ensure access to the high-quality data, a series of stringent quality control measures were instituted. Trimmomatic (version 0.36) was judiciously configured with specific parameters, including a sliding window strategy set at 50 bp, an average quality threshold of 20, and a minimum sequence length requirement of 120 bp. Furthermore, sequences containing ambiguous bases (‘N’) were systematically eliminated using Pear (version 0.9.6) [25]. Subsequent data processing involved merging sequences from both ends to produce fasta sequences. This was achieved by employing Flash and Pear with specific settings, including a minimum overlap of 10 bp and a mismatch rate of 0.1. A critical step in this process involved identifying and removing chimeric sequences, which was accomplished by comparing sequences against the Gold OnLine Database (GOLD) using the UCHIME algorithm. Simultaneously, any sequences that remained unaligned with available databases were removed using a de novo approach [26]. Short sequences failing to meet the stringent quality criteria were rigorously eliminated, yielding a final set of high-quality sequences constituting clean reads.

The subsequent clustering of these clean reads into operational taxonomic units (OTUs) was achieved using the UPARSE algorithm within Vsearch (version 2.7.1). For each OTU, the most frequently occurring sequence was thoughtfully selected as the representative sequence [27]. To assign species classification information to each OTU, we subjected the representative sequences to comprehensive analysis using the RDP Classifier algorithm (version 2.2) [28] and the SILVA database [29]. This meticulous process enabled us to perform community annotation at multiple taxonomic levels, including kingdom, phylum, class, order, family, and genus. After the standardization of the sequence counted to the lowest sample number, we computed alpha diversity metrics (Chao1, Shannon, Simpson, and observed species) using QIIME (version 1.8.0). To assess intergroup alpha index variability, we employed the Kruskal–Wallis test in R (version 4.0.2) [30]. Principal coordinate analysis (PCoA) based on Bray–Curtis dissimilarity matrices, implemented using QIIME, was used to examine variations in bacterial communities among the experimental groups. The linear discriminant analysis effect size (LEfSe, LDA > 3) was employed to pinpoint significant bacteria within the three experimental groups [30]. Notably, differences in alpha diversity and the relative abundance of taxa at the phylum, family, and genus levels were assessed utilizing the Kruskal–Wallis method in R. The ensuing results were meticulously visualized through a range of graphical techniques facilitated by the “ape”, “ggplot2”, “limma”, “ggplot2”, “ggtree”, and “igraph” packages in R.

### 2.7. Statistical Analysis

First of all, the error and variances of all the experimental data were detected. The error of all data obeyed an NIID (0, σ^2^) normal distribution, and the variances of each group were equal. Statistical analyses of various parameters, including BW, ADG, DMI, and feed efficiency of the lamb groups, as well as of samples of intestinal tissue, were carried out using SAS software (version 9.2, SAS Inst. Inc., Cary, NC, USA) with one-way ANOVA followed by Duncan’s multiple comparison tests. The statistical model used for this analysis was:

Y_i_ = µ + T_i_ + E_i_ (1)

In this model, Y_i_ represents the dependent variable, µ is the overall mean, T_i_ is the treatment effect, and E_i_ is the error term. Multiple comparisons of means among different treatments were conducted using Duncan’s multiple comparison tests. 

To assess the concentrations of serum antibodies, a two-way ANOVA in the PROC GLM of SAS was used. This allowed us to examine the impact of the effect of treatment, day, and the interaction between treatment and day on the serum antibody concentrations. The statistical model used for this analysis was:

Y_ij_ = µ + T_i_ + D_j_ + TD_ij_ + E_ij_ (2)

where Y_ij_ represents the dependent variable, µ is the overall mean, T_i_ is the treatment effect, D_j_ is the age effect, TD_ij_ is the interaction between treatment and age, and E_ij_ is the error term. All reported data were presented as means with a significance level of 0.05.

## 3. Results

### 3.1. Feed Intake and Growth Performance

As shown in Table 2, the birth weight of lambs among the three groups was significantly different (*p* < 0.05), but there was no difference in the BW of lambs among the three groups after 21 days of age (*p* > 0.05). No differences were observed in the ADG of the three groups lambs pre-weaning (*p* > 0.05). However, after weaning, on day 28, the ADG of lambs in the EW group and AP group was significantly lower than that of lambs in the ER group (*p* < 0.001). No significant differences were observed in the starter feed DMI of lambs among the three groups throughout the experiment (*p* > 0.05). Before weaning, the feed efficiencies of the ER group and AP group were significantly lower than that of the EW group (*p* < 0.05). After weaning, on day 28, compared to the ER group, the feed efficiency of the EW group and AP group decreased significantly (*p* < 0.001). No significant differences were noted in the ADG and feed efficiency between the EW group and AP group; however, compared to the EW group, an upward trend was observed in the AP group (*p* = 0.094 and 0.239, respectively).

### 3.2. Serum Antibodies Concentrations

Table 3 shows the immune response among the groups. Significant interactions were noted between treatment and age for IgG (F = 7.195, *p* < 0.001), IgA (F = 11.817, *p* < 0.001), and IgM (F = 11.488, *p* < 0.001) levels. The IgG concentration was lower in the ER group than that in the other two groups; however, this phenomenon was reversed on the third day after weaning (day 31). Moreover, the concentration of IgG on days 28 and 35 and thereafter was unaffected by the treatment. The concentrations of IgA and IgM followed the same trend; their concentration in the ER group was higher than those in the EW and AP groups on days 21 and 28, but lower on day 31. However, no difference was observed in the IgA and IgM levels among the three groups on day 35 and thereafter.

### 3.3. Small Intestinal Morphology

**Figure 1:**
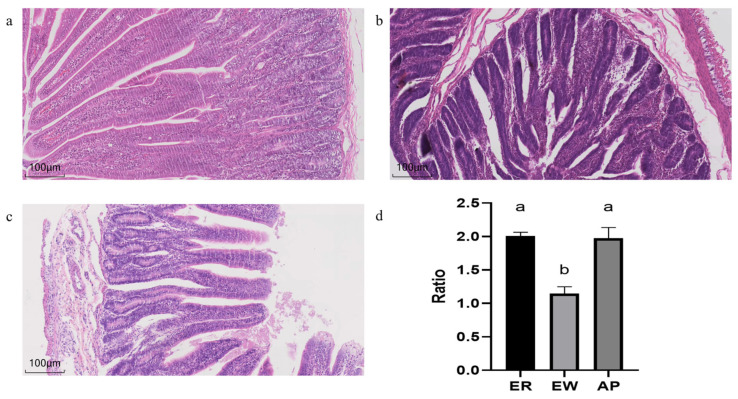
Histological changes in the jejunum of lambs. (**a**–**c**) HE-stained sections of the ER group, EW group, and AP group, respectively. (**d**) The V/C ratio of the ER group, EW group, and AP group. Different lowercase letters stand for significant differences (*p* < 0.05).

As shown in Figure 1, different treatments affected the morphology and structure of the jejunum of the lambs. Compared to the ER group, in the EW group, the villi were shortened, the crypt depth increased, and V/C was significantly reduced (*p* < 0.01). However, pre-feeding for the AP lambs restored the V/C downregulation (*p* < 0.001).

### 3.4. Taxonomic Composition of Gastrointestinal Bacteria

**Figure 2:**
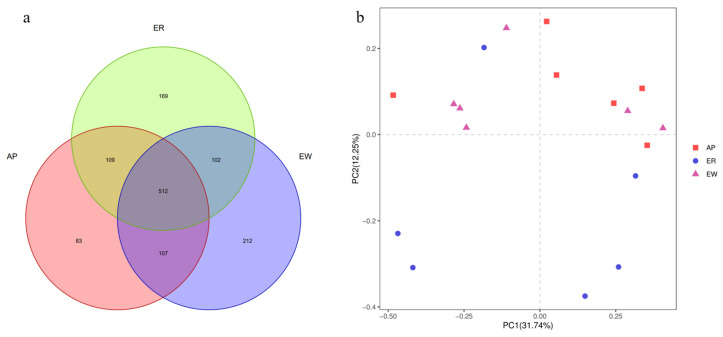
Response of intestinal microbiota from lambs among the three groups. (**a**) Composition of intestinal microbiota from lambs among the three groups (OTU level analysis). (**b**) PCoA analysis of intestinal microbiota from lambs among the three groups. ER, ewe-reared group; EW, early weaning group; AP, antimicrobial-peptide-fed group.

The gradual stabilization of the OTU rank curve chart (Supplementary Figure S1) suggests that the test samples had good coverage. In total, 1,307,195 high-quality reads were obtained, with an average of 72,622 reads per sample, and the sequence lengths ranged from approximately 380 to 440 bp. Sequence coverage was considered sufficient, as indicated by the Good’s coverage (>0.98) for all samples. Based on 97% nucleotide sequence identification, we identified a total of 1,294 OTUs, with an average of 331 ± 98 OTUs per sample. Specifically, the ER group had 892 OTUs, the EW group had 933 OTUs, and the AP group had 811 OTUs (Figure 2a). Among these, 512 OTUs (39.57% of the total) were shared among samples from different groups. Notably, 614 OTUs (47.45%) were shared between the ER group and EW group, 614 OTUs (47.99%) were common between the ER group and AP group, and 619 OTUs (47.84%) were shared between the EW group and AP group. These findings indicate differences in OTU composition among the different groups.

To assess the richness and diversity of the intestinal bacterial microbiota within the three groups, we employed Chao1, observed_species, PD_whole_tree, Shannon, and Simpson indices. However, no significant differences in alpha diversity were observed among the three groups (*p* > 0.05, Table 4). These results suggest that the lambs in the three groups had similar gastrointestinal microbiota structures and compositions.

Further analysis using PCoA plots of bacterial structure profiles (Figure 2b) revealed distinct clustering among the different treatments. The Bray–Curtis matrices indicated there were significant segregation and dissimilarities at the OTU level among the three groups (PERMANOVA; *p* < 0.01). These findings imply that the gastrointestinal microbiota community structure underwent changes due to weaning stress and pre-weaning feed with AP, highlighting the potential role of microbiota in the development of weaning stress.

**Figure 3:**
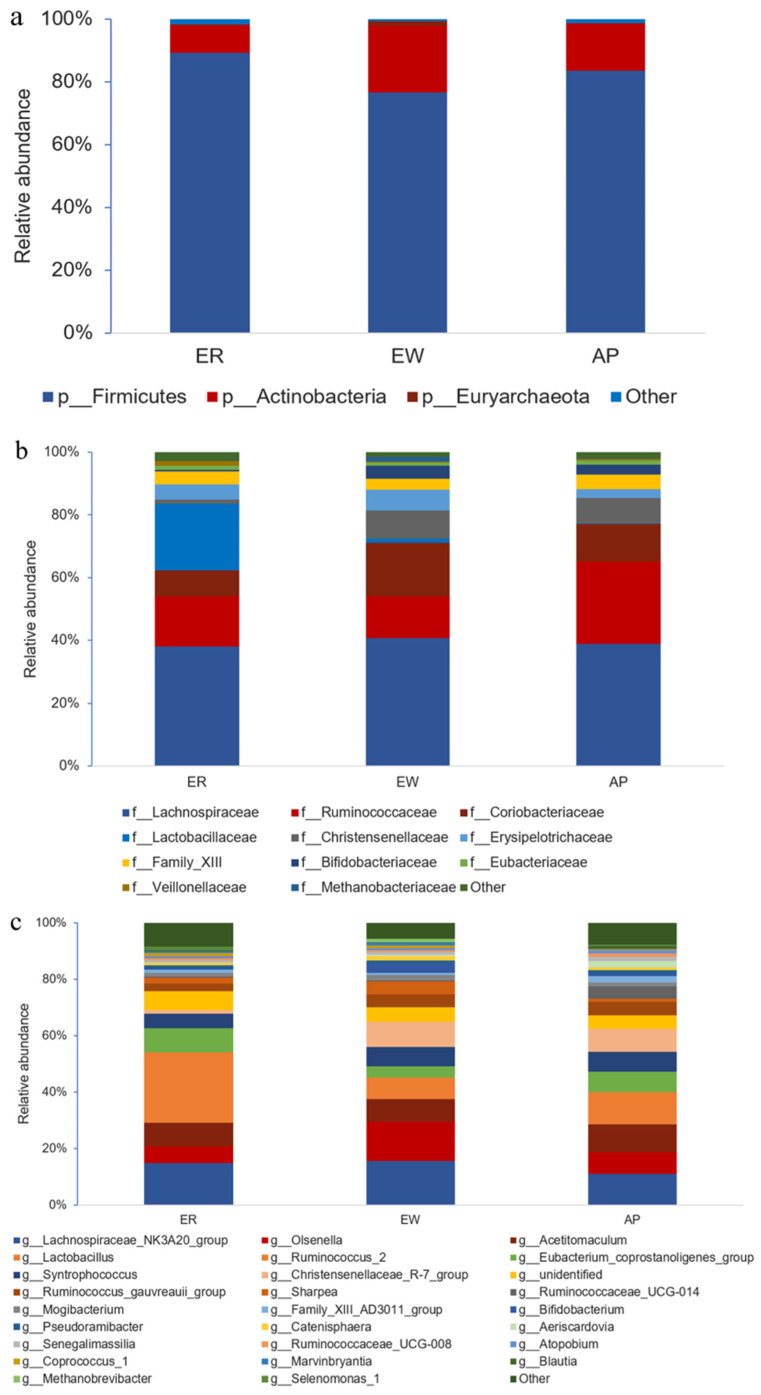
Compositional profiles of intestinal microbiota from lambs among the three groups. (**a**) Phylum level, (**b**) family level, and (**c**) genus level. ER, ewe-reared group; EW, early weaning group; AP, antimicrobial-peptide-fed group.

Furthermore, we performed bacterial identification of 18 samples at different levels. At the phylum level, the dominant bacteria in lambs across all samples were the same, mainly Firmicutes (83.15%) and Actinobacteria (15.93%) (Figure 3a and Supplementary Table S1). The relative abundance of Firmicutes was significantly lower in the EW group compared to the ER group; however, there was no significant difference between the AP group and the other two groups (*p* < 0.05). Interestingly, the trend in the relative abundance of Actinobacteria was opposite to that of Firmicutes (*p* = 0.052). At the family level, *Lachnospiraceae* (39.26%) was the most abundant taxon, followed by *Ruminococcaceae* (18.48%) and *Coriobacteriaceae* (12.38%) across all samples; other families included *Lactobacillaceae* (7.60%), *Christensenellaceae* (5.87%), *Erysipelotrichaceae* (4.69%), *Family_XIII* (4.12%), *Bifidobacteriaceae* (2.96%), and *Eubacteriaceae* (1.22%) (Figure 3b and Supplementary Table S2). Notably, compared to the ER group, the relative abundance of *Lactobacillaceae* in the EW and AP groups decreased significantly (*p* < 0.01), but no significant difference between EW and AP groups was observed, suggesting that *Lactobacillaceae* was related to weaning stress. In contrast, the relative abundance of *Coriobacteriaceae* and *Bifidobacteriaceae* in the EW group increased rapidly compared to that in the ER group, whereas there was no significant difference between the AP group and the other two groups (*p* = 0.108 and 0.093, respectively). At the genus level, the dominant genera across the three groups were *Lachnospiraceae_NK3A20_group* (13.77%), *Olsenella* (9.18%), *Acetitomaculum* (8.73%), *Lactobacillus* (7.60%), and *Ruminococcus_2* (7.07%) (Figure 3c and Supplementary Table S3). Compared to the ER group, the relative abundance of *Lactobacillus* in the EW and AP groups decreased significantly (*p* < 0.01), whereas the variation trend in the relative abundance of *Bifidobacterium* was the opposite (*p* = 0.074). Moreover, the relative abundance of *Aeriscardovia* in the AP group was significantly higher than that in the other two groups (*p* < 0.01), indicating that *Aeriscardovia* is associated with the efficacy of AP.

**Figure 4:**
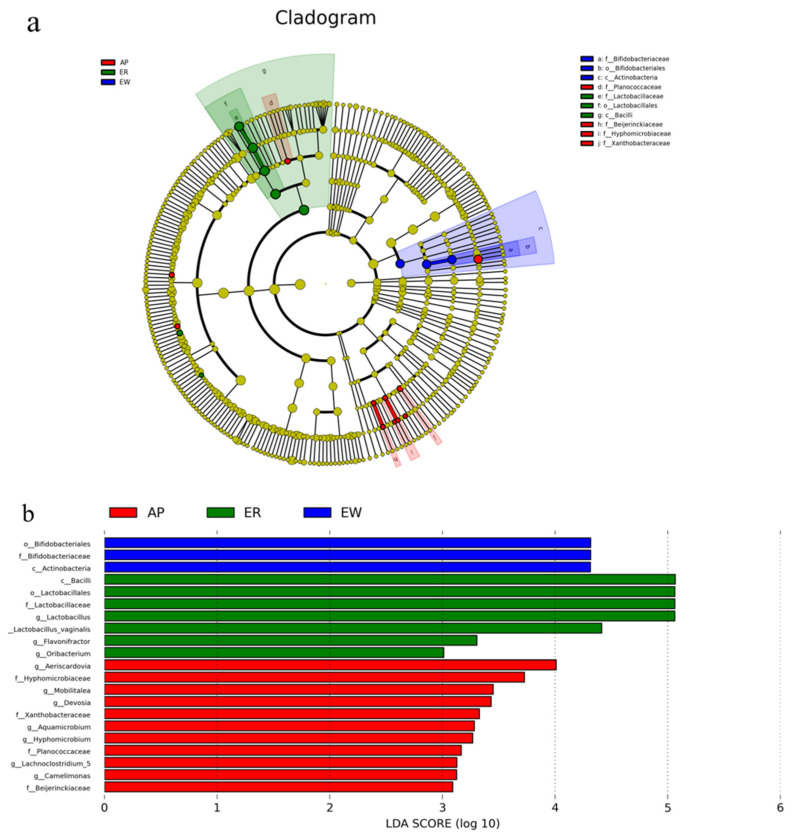
LEfSe analysis of intestinal microbiota from lambs among the three groups. (**a**) Histogram of linear discriminant analysis scores based on classification information. (**b**) Linear discriminant analysis effect size cladogram based on classification information. ER, ewe-reared group; EW, early weaning group; AP, antimicrobial-peptide-fed group.

To better understand the dominance of specific bacteria within the three groups, we used the LEfSe method (Figure 4). *Lactobacillaceae*, *Lactobacillales*, and *Bacilli* were enriched in the ER group. *Bifidobacteriaceae*, *Bifidobacteriales*, and *Actinobacteria* were overrepresented in the EW group. *Planococcaceae*, *Beijerinckiaceae*, *Hyphomicrobiaceae*, and *Xanthobacteraceae* were abundant in the AP group.

### 3.5. Growth Performance and Serum Immune Indicators Correlate with Bacterial Communities

**Figure 5:**
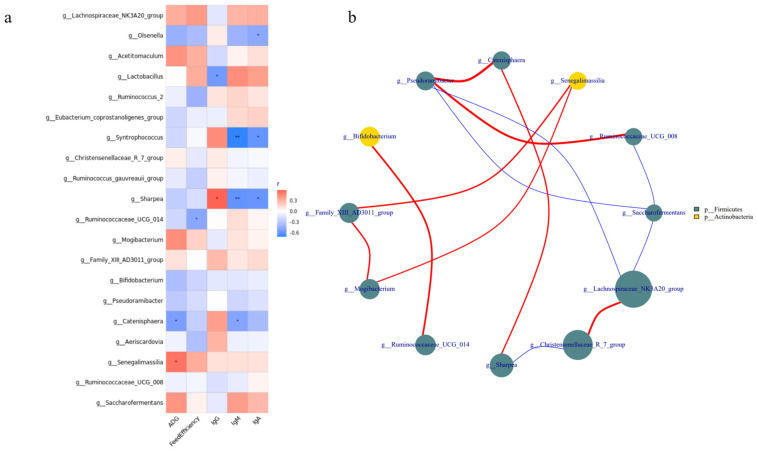
Correlation analysis of intestinal microbiota from lambs among the three groups. (**a**) Correlation between the top 20 relative abundances of genera, growth performance, and serum immune indicators. (**b**) Interaction among the top 20 relative abundances at the genus level. Red represents a positive correlation and blue a negative correlation. The number represents the correlation coefficient. * represents *p* < 0.05, ** represents *p* < 0.01. The size and color of the circle represent the relative abundance of the respective genus and the phylum.

The correlation between the relative abundance of the top 20 bacterial genera, growth performance, and serum immune indicators (Figure 5a) revealed that the relative abundances of *Lactobacillus*, *Ruminococcaceae_UCG-014*, *Catenisphaera*, and *Senegalimassilia* were correlated with growth and immunity (*p* < 0.05). For example, the relative abundance of *Lactobacillus* was positively correlated with IgG levels and negatively correlated with IgM and IgA levels. The relative abundance of *Ruminococcaceae_UCG-014* was negatively correlated with feed efficiency. In addition, the relative abundance of *Catenisphaera* was negatively correlated with ADG, whereas that of *Senegalimassilia* was positively correlated with ADG. Interestingly, we found a significant positive correlation between *Ruminococcaceae_UCG-014* and *Bifidobacterium* (*p* < 0.05; Figure 5b).

## 4. Discussion

Early weaning stress can lead to decreased feed intake, impaired intestinal morphology, altered microbiota structure, and imbalanced homeostasis in lambs [12,31,32]. This subsequently leads to pathogen invasion and intestinal inflammation, resulting in increased rates of diarrhea and stunted growth [32]. Understanding how to regulate the intestinal homeostasis of early-weaned lambs by regulating the intestinal microbiota is necessary to alleviate intestinal injury and improve the production performance of early-weaned lambs. We described the effects of early weaning on lamb growth performance, the serum immune status, and jejunal microbial composition and explored the mechanism by which yeast peptides alleviate lamb weaning stress by pre-feeding 1 g/day before weaning.

Our results showed that weaning stress led to a decrease in ADG and feed efficiency and reduced the production performance of lambs. Although feeding yeast peptide did not significantly alleviate this phenomenon, the ADG and feed efficiency of the AP group improved compared to those of the EW group. ZHANG et al. [33] also found that antimicrobial peptides can increase ADG and feed efficiency of the poultry. Previous studies found that IgG accounts for 75% of the total immunoglobulins, is the most persistent and important antibody in the primary immune response, and can effectively remove the antigens [34]. Its concentration is positively correlated with animal health [35]. IgA can inhibit microbial attachment, slow viral reproduction, and resist pathogen invasion [36]. IgM has strong effects on sterilization, bacteriolysis, phagocytosis, and agglutination, all of which play important roles in early immune defense [37]. In a recent study, the blood biochemical indexes of Hu sheep aged 1, 2, 3, 6, and 12 months were measured, and the normal distribution range of serum antibody concentrations was determined (IgG: 17.51 ± 1.33 g/L, IgM: 0.97 ± 0.15 g/L, and IgA: 0.41 ± 0.05 g/L) [38]. In our study, the serum antibody concentrations mostly fell within this established range, with only a slightly elevated IgA level. Despite these fluctuations, our findings, consistent with those of WANG et al. [32], revealed no significant differences in the serum immune capacity of the three groups of lambs throughout the entire experiment. However, compared with the ER group, in the EW group, after weaning (day 31), the concentration of IgG decreased significantly, whereas the concentrations of IgA and IgM increased significantly. This indicated that the immune status of lambs underwent changes. However, further longer-term experiments are needed to obtain the relevant data support. Since the immune system of young animals is not fully developed, they primarily rely on the intake of immunoglobulins from breast milk for immunization [39]. Consequently, after weaning, the supplement of maternal antibodies ceases, resulting in a decrease in the immune function and a weakened ability to protect against pathogen invasion in lambs [40]. The results showed that lambs weaned at an early stage, compared to those raised by ewes, were more susceptible to decreased immunological functions, external bacterial infections, and a compromised overall health.

The intestinal epithelial barrier is the first line of defense that limits the invasion of bacteria and viruses, and a damaged barrier function is the main cause of intestinal inflammation [41]. An intact intestinal structure is key to normal intestinal barrier function. A decrease in intestinal villus height and an increase in crypt depth are markers of intestinal barrier function damage [42]. CUI et al. [7] found that early weaning stress can induce intestinal villus damage. Antimicrobial peptides can effectively inhibit harmful intestinal bacteria, regulate intestinal inflammation, and improve the intestinal antioxidant capacity to maintain the normal structure and morphology of the intestinal tract [43]. The results showed that weaning stress resulted in atrophy of jejunal villi, proliferation of crypts, and a decrease in V/C; however, the yeast peptide significantly increased V/C. YOON et al. [44] added antimicrobial peptide A3 to the diet of piglets to increase V/C, which is consistent with the results of our research.

The intestinal tract is the largest bacterial bank in mammals, and a mutually beneficial symbiosis exists between probiotics and hosts, which not only secretes bacteriocin, prevents the attachment of pathogenic bacteria, and inhibits the growth of pathogenic bacteria to improve immunity, but also provides nutrients to the body. It can also protect the mucous membrane, regulate machinery homeostasis, and ensure intestinal health [45,46]. Firmicutes and Actinobacteria were the main jejunal microbiota in all three lamb groups. Compared to the ER group, the abundance of Firmicutes in the EW group decreased significantly, but no significant difference was noted between the AP group and the other two groups. In other words, pre-feeding with yeast peptides alleviated the decrease in jejunal Firmicutes abundance caused by weaning stress. As Firmicutes play an important role in energy conversion [47], our results showed that a decrease in their abundance might be the key factor leading to the low feed conversion rate of early weaning lambs. Furthermore, *Lactobacillus* is an anaerobic Gram-positive bacterium that can protect the intestinal barrier by preventing pro-inflammatory factors from destroying intestinal tight junctions, inducing the expression of mucin MUC2, and secreting antibacterial substances to inhibit the adhesion of pathogenic *Escherichia coli* [48,49]. We found that weaning stress resulted in a significant decrease in the abundance of *Lactobacillus* in lambs, resulting in the destruction of the intestinal mucosal barrier and damage to intestinal morphology and structure. In addition, the results of the correlation analysis showed that the relative abundance of *Lactobacillus* was positively correlated with the concentration of IgG, and the intestinal immunity of the lambs was downregulated. We speculated that weaning stress could downregulate the relative abundance of *Lactobacillus*, further compromise the protective function of the intestinal mucosal barrier, and diminish intestinal immunity. Certainly, further research is needed to explore the correlation between the related proteins expression and the abundance of *Lactobacillus*. Both *Aeriscardovia* and *Senegalimassilia* belong to Actinobacteria, which is involved in regulating intestinal barrier integrity [50], immune function [51], and metabolism [52]. Notably, as a popular family of Actinobacteria, *Bifidobacterium* has been widely researched as a kind of intestinal probiotic. *Aeriscardovia* and *Senegalimassilia* are coordinated with *Bifidobacterium*. Numerous experiments have demonstrated its ability to produce large amounts of short-chain fatty acids (SCFAs) and lactate through carbohydrate fermentation, thereby regulating the intestinal epithelial cell metabolism and the expression of MUC2 [50,53,54]. Simultaneously, *Bifidobacterium* can enhance antimicrobial [55] and anti-inflammatory [56] capabilities and fortify the protective function of the intestinal epithelial barrier [50]. Moreover, DUCA et al. [57] discovered a negative correlation between the relative abundance of *Bifidobacterium* and intestinal permeability. It also boosts immunity by regulating the differentiation and expression of macrophages [58], T-cells [59], and inflammatory cytokines [60]. *Bifidobacterium* participates in the digestion and metabolism of intestinal organic substances through the production of various enzymes [61]. Additionally, the synergistic anti-disease effect of *Bifidobacterium* and *Lactobacillus* has also been reported [50]. It was found that the relative abundance of *Aeriscardovia* decreased significantly under heat stress, along with a decreased growth performance, intestinal damage, and immune dysfunction in piglets [62]. This suggested that there is a correlation between *Aeriscardovia* and stress reactions. *Senegalimassilia*, initially isolated from healthy human feces in 2020 [63], is notably reduced in patients with an acute fatty liver [64]. Under our experimental conditions, the relative abundance of both types of *Bifidobacterium* increased following feeding with yeast peptides, which mitigated the decline in daily weight gain of weaned lambs. We deduced that yeast peptides might enhance the intestinal metabolism and immune function, ultimately improving the growth performance of early weaning lambs by increasing their relative abundance. *Ruminococcaceae_UCG-014*, akin to *Bifidobacterium*, belongs to *Ruminococcaceae*, which has the capacity to produce SCFAs, serving as an energy source for enterocytes. However, unlike *Bifidobacterium*, which primarily produces acetic acid, *Ruminococcaceae* mainly produces butyric acid [50]. In addition to degrading complex polysaccharides to produce butyric acid, WANG et al. [65] reported a significant downregulation in *Ruminococcaceae_UCG-014* during inflammation. Conversely, XING et al. [66] found that the relative abundance of *Catenisphaera* increased significantly under heat stress. Interestingly, in psychological stress research, a similar increase in *Catenisphaera* abundance under stress conditions disrupted the intestinal barrier function [67]. Consistent with our findings, the relative abundance of *Ruminococcaceae_UCG-014* increased, but *Catenisphaera* decreased after feeding yeast peptides compared to the EW group. This could be attributed to yeast peptides being rich in amino acids, which can serve as fermentation substrates for intestinal microbiota, thereby increasing the abundance of bacteria that preferentially utilize these amino acids. Meanwhile, yeast peptides possess antimicrobial properties, inhibiting the proliferation of harmful bacteria and reducing competition for beneficial bacteria, leading to an increase in their abundance. Additionally, their immunomodulatory effects may impact microbiota by modulating the host immune system. In summary, we hypothesize that yeast peptides, owing to their nutritional origin and broad-spectrum antimicrobial and immunomodulatory properties, have the capacity to modulate the healthy intestinal microbiota of early weaning lambs. This modulation may extend to regulating the intestinal morphology and structure of lambs, enhancing their immunity, suppressing inflammation, facilitating nutrient digestion and metabolism, and ultimately improving the growth performance of early weaning lambs.

## 5. Conclusions

In this study, we compared the growth performance, serum antibody concentrations, jejunal histomorphology, and jejunal bacterial composition of ewe-reared lambs, early weaning lambs, and early weaning lambs supplemented with yeast peptides. Our findings revealed that early weaning resulted in a reduced growth performance and immunocompetence, damage to intestinal morphology, and disruption of the intestinal microbiota balance in lambs. Importantly, the supplementation of yeast peptides alleviated the reduction in ADG and the intestinal damage in lambs induced by early weaning. This effect may be associated with changes in the abundance of genera *Lactobacillus*, *Ruminococcaceae_UCG-014*, *Senegalimassilia*, and *Catenisphaera*. These findings provide new insights into mitigating the effects of early weaning stress in lambs, though further research is required to validate the identified bacteria and clarify their interactions with the host.

## Figures and Tables

**Table 1:** Ingredients and chemical composition of the starter feed.

Item	Content
Ingredients	(% of feeding basis)
Corn	40.0
Soybean meal	20.0
Whey powder	20.0
Alfalfa hay	16.0
Premix ^(1)^	4.0
Total	100.0
Nutrient levels ^(2)^	(% dry matter)
NEL/(MJ/kg)	11.59
CP	17.15
EE	3.87
Ash	9.10
NDF	20.61
ADF	11.29
Ca	1.45
P	0.57

^(1)^ One kilogram of premix contained the following minerals and vitamins: vitamin A, 12,000 IU; vitamin D, 2000 IU; vitamin E, 30 IU; Fe, 64 mg; Cu, 12 mg; Mn, 56 mg; Zn, 60 mg; I, 1.2 mg; Se, 0.4 mg; Co, 0.4 mg; NaCl, 6 g. ^(2)^ NEL values were calculated according to NRC (2007); others were measured by laboratory analysis of the total mixed ration.

**Table 2:** Growth performance and feed intake of lambs with different treatments (n = 18 per group).

Items	Treatment			SEM	*p*-Value
	ER	EW	AP		
BW, kg					
1 d	3.32 ^b^	3.59 ^ab^	3.81 ^a^	0.08	0.045
21 d	6.76	6.85	7.43	0.18	0.275
28 d	8.13	9.03	8.44	0.22	0.262
35 d	9.53	9.49	9.24	0.24	0.870
ADG, g/d					
Pre-weaning	171.83	194.25	165.48	7.24	0.673
Post-weaning	199.21 ^a^	65.24 ^b^	113.89 ^b^	13.73	<0.001
DMI ^(1)^, g/d					
Pre-weaning	34.44	23.41	46.43	6.52	0.407
Post-weaning	130.08	128.89	164.92	15.20	0.612
Feed Efficiency ^(2)^,%					
Pre-weaning	6.65 ^b^	11.73 ^a^	6.24 ^b^	0.72	0.002
Post-weaning	1.65 ^a^	0.43 ^b^	0.73 ^b^	0.12	<0.001

ER, ewe-reared group; EW, early weaning group; AP, antimicrobial-peptide-fed group; BW, body weight; ADG, average daily gain; DMI, dry matter intake of starter feed. Different superscript letters in the same row denote significant differences (*p* < 0.05). ^(1)^ DMI represents the group DMI, which was calculated by the average daily feed intake of lambs in each pen. ^(2)^ Feed efficiency represents the group feed efficiency, which was calculated as ADG/DMI.

**Table 3:** Serum antibodies concentrations of lambs with different treatments (n = 18 per group).

Items	Treatment			SEM	*p*-Value		
	ER	EW	AP		T	D	T × D
IgG, g/L							
21 d	16.28 ^b^	17.56 ^a^	17.43 ^a^	0.18	0.001		
28 d	16.72	17.00	16.70	0.12	0.529		
31 d	18.25 ^a^	16.85 ^b^	16.78 ^b^	0.21	0.001		
35 d	15.99	16.39	16.68	0.20	0.394		
Overall	16.81	16.95	16.89	0.10	0.726	<0.001	<0.001
IgA, g/L							
21 d	0.69 ^a^	0.62 ^c^	0.65 ^b^	0.01	<0.001		
28 d	0.70 ^a^	0.63 ^b^	0.64 ^b^	0.01	0.001		
31 d	0.55 ^b^	0.68 ^a^	0.66 ^a^	0.01	<0.001		
35 d	0.73	0.66	0.70	0.01	0.172		
Overall	0.67	0.65	0.66	0.01	0.145	<0.001	<0.001
IgM, g/L							
21 d	1.02 ^a^	0.92 ^b^	0.94 ^b^	0.01	0.001		
28 d	1.00 ^a^	0.93 ^b^	0.96 ^ab^	0.01	0.041		
31 d	0.79 ^b^	1.01 ^a^	0.97 ^a^	0.02	<0.001		
35 d	1.07	0.97	1.02	0.02	0.209		
Overall	0.97	0.96	0.97	0.01	0.598	<0.001	<0.001

ER, ewe-reared group; EW, early weaning group; AP, antimicrobial-peptide-fed group; T, treatment; D, day. Different superscript letters in the same row denote significant differences (*p* < 0.05).

**Table 4:** Alpha diversity of gastrointestinal microbiota in each group. (*n* = 6 per group.)

Items	Groups			SEM	*p*
	ER	EW	AP		
Chao1	470.31	408.99	441.47	27.26	0.682
Observed_species	333.82	319.98	340.48	23.02	0.941
PD_whole_tree	30.59	30.79	31.53	1.96	0.981
Shannon	4.69	4.54	4.74	0.15	0.854
Simpson	0.89	0.89	0.91	0.13	0.850

ER, ewe-reared group; EW, early weaning group; AP, antimicrobial-peptide-fed group.

## Data Availability

The 16S rDNA gene sequencing reads were deposited in the Genome Sequence Archive in the BIG Data Center under the accession number PRJNA981228.

## Supplementary Materials

The following supporting information can be downloaded at: https://www.mdpi.com/article/10.3390/microorganisms11102472/s1, Table S1: Comparison of intestinal microbiota at the phylum level; Table S2: Comparison of intestinal microbiota at the family level; Table S3: Comparison of intestinal microbiota at the genus level; Figure S1: Specaccum species accumulation curve.
