# Supplementary material for: Yeast Peptides Improve the Intestinal Barrier Function and Alleviate Weaning Stress by Changing the Intestinal Microflora Structure of Weaned Lambs
Source: Microorganisms. 2023 Oct 1;11(10):2472. doi: 10.3390/microorganisms11102472 (PMC10608930; doi:10.3390/microorganisms11102472)
Supplement: Supplementary file 1 [file microorganisms-11-02472-s001.zip › microorganisms-2563947-supplementary.pdf]

Supplementary Table S1. Comparison of intestinal microbiota at the phylum level.

Phylum	Groups			SEM	*p*
	ER	EW	AP		
Firmicutes	89.31[a]	76.64[b]	83.50[ab]	2.16	0.045
Actinobacteria	8.89	21.23	14.96	2.14	0.052
Euryarchaeota	0.09	1.43	0.20	0.44	0.408
Bacteroidetes	0.51	0.17	0.44	0.14	0.604
Tenericutes	0.65	0.02	0.26	0.20	0.470
Cyanobacteria	0.03	0.30	0.24	0.08	0.353
Verrucomicrobia	0.20	0.05	0.05	0.06	0.573

ER, ewe-reared group; EW, early-weaning group; AP, early-weaning and pre-feeding 1 g/d yeast peptide group.

Different superscript letters in the same row denote significant differences ($p < 0.05$).

Supplementary Table S2. Comparison of intestinal microbiota at the family level.

Family	Groups			SEM	*p*
	ER	EW	AP		
Lachnospiraceae	38.11	40.79	38.89	3.01	0.940
Ruminococcaceae	15.92	13.29	26.24	2.96	0.171
Coriobacteriaceae	8.26	17.08	11.80	1.74	0.108
Lactobacillaceae	21.25^{a}	1.30^{b}	0.26^{b}	3.37	0.007
Christensenellaceae	1.41	8.10	8.10	3.26	0.611
Erysipelotrichaceae	4.70	6.55	2.81	1.32	0.538
Family_XIII	4.17	3.56	4.62	0.74	0.858
Bifidobacteriaceae	0.61	5.13	3.15	0.69	0.093
Eubacteriaceae	1.20	1.06	1.39	0.39	0.947
Veillonellaceae	1.52	0.32	0.53	0.47	0.572
unidentified	0.86	0.45	0.59	0.22	0.771
Methanobacteriaceae	0.09	1.43	0.20	0.44	0.408
Bacillaceae	0.75	0.27	0.24	0.17	0.416
Prevotellaceae	0.37	0.12	0.39	0.12	0.601
Streptococcaceae	0.89	0.27	0.10	0.08	0.614

ER, ewe-reared group; EW, early-weaning group; AP, early-weaning and pre-feeding 1 g/d yeast peptide group.

Different superscript letters in the same row denote significant differences ($p < 0.05$).

Supplementary Table S3. Comparison of intestinal microbiota at the genus level.

Genus	Groups			SEM	*p*
	ER	EW	AP		
Lachnospiraceae_NK3A20_group	14.74	15.58	10.98	3.16	0.836
Olsenella	5.98	13.95	7.60	1.79	0.158
Acetitomaculum	8.38	7.91	9.91	1.55	0.874
Lactobacillus	21.25[a]	1.30[b]	0.26[b]	3.37	0.007
Ruminococcus_2	3.64	6.35	11.22	2.07	0.336
Eubacterium_coprostanoligenes_group	8.65	4.07	7.14	1.24	0.324
Syntrophococcus	5.11	6.83	7.18	1.68	0.879
Christensenellaceae_R-7_group	1.39	8.99	8.02	3.26	0.612
unidentified	6.53	5.00	4.88	0.76	0.642
Ruminococcus_gauvreauii_group	2.81	4.65	4.73	0.72	0.492
Sharpea	2.13	4.57	1.22	1.08	0.446
Ruminococcaceae_UCG-014	0.31	0.34	4.22	0.82	0.073
Mogibacterium	1.29	1.91	1.34	0.45	0.837
Family_XIII_AD3011_group	1.27	0.81	2.39	0.32	0.124
Bifidobacterium	0.26	3.30	0.70	0.60	0.074
Pseudoramibacter	1.19	1.06	1.38	0.39	0.948
Catenisphaera	0.76	1.58	0.91	0.32	0.562
Aeriscardovia	0.24[b]	0.49[b]	2.33[a]	0.31	0.004
Senegalimassilia	0.60	0.89	1.46	0.33	0.581
Ruminococcaceae_UCG-008	0.80	0.64	1.29	0.42	0.826

ER, ewe-reared group; EW, early-weaning group; AP, early-weaning and pre-feeding 1 g/d yeast peptide group.

Different superscript letters in the same row denote significant differences ($p < 0.05$).

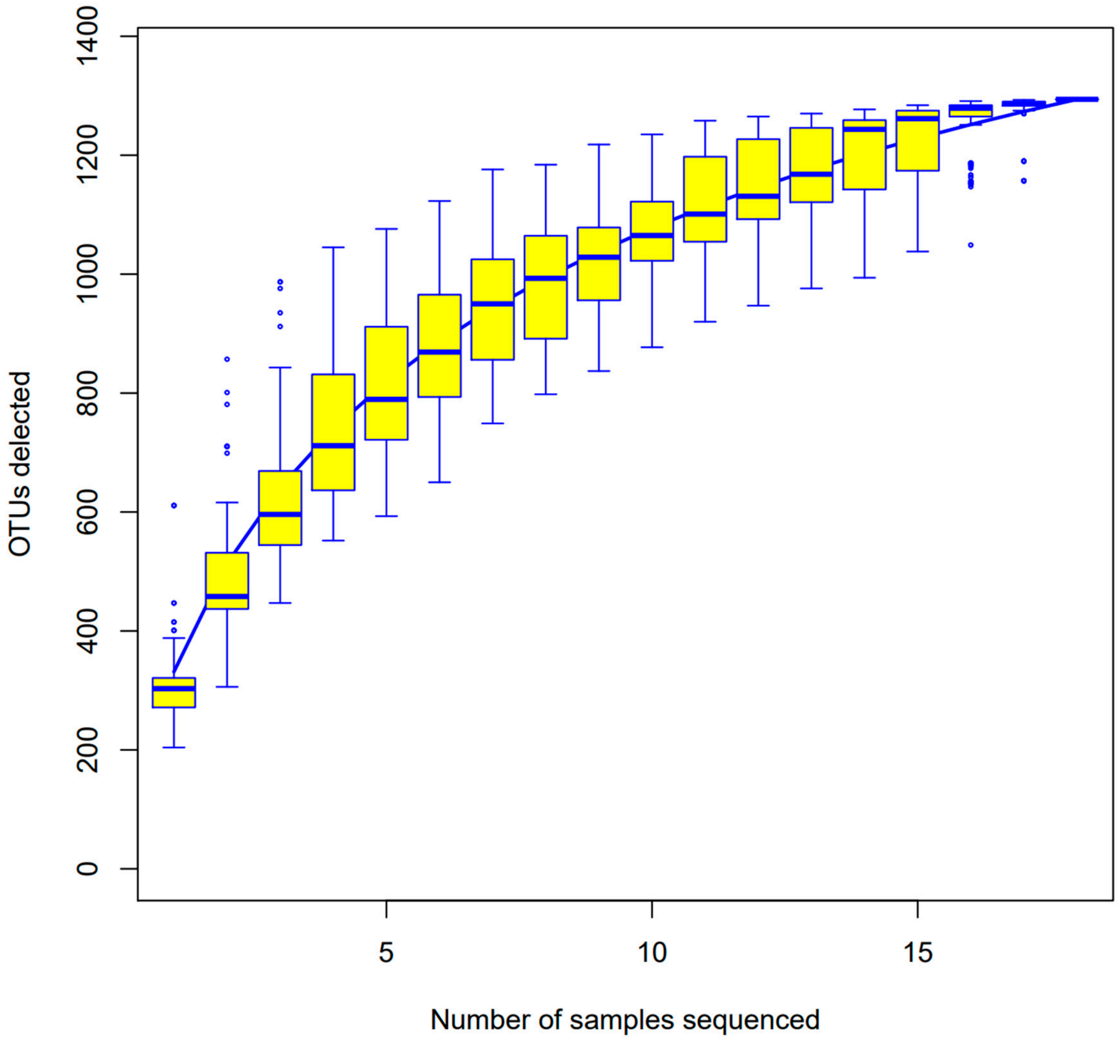

Supplementary Figure S1. Specaccum species accumulation curve.
